# Acute Pancreatitis Caused by Isotretinoin

**DOI:** 10.7759/cureus.8710

**Published:** 2020-06-20

**Authors:** Muddasir Ashraf

**Affiliations:** 1 Hospital Medicine, UnityPoint Health Trinity Rock Island, Rock Island, USA

**Keywords:** isotretinoin, pancreatitis, acne, hypertriglyceridemia, lipid, dermatological, drug, retinoid, metabolism

## Abstract

Isotretinoin, a retinoid derivative, is used widely as a treatment for severe acne and other dermatological conditions. Its effect on lipid metabolism, especially the induction of hypertriglyceridemia, is well documented. There are some case reports in the literature about drug-induced pancreatitis secondary to isotretinoin. We describe another interesting case of acute pancreatitis related to severe hypertriglyceridemia due to isotretinoin.

## Introduction

Acute pancreatitis is characterized by inflammation of the pancreas manifested clinically by abdominal pain and elevated levels of serum pancreatic enzymes [1]. The underlying pathogenesis of acute pancreatitis is not entirely understood. Nevertheless, several conditions are known to cause this disorder, with gallstones and chronic alcohol abuse accounting for two-thirds of the cases in the United States. This list will undoubtedly continue to grow, and the number of cases described as "idiopathic" will decrease as we understand it better. The reported annual incidence of acute pancreatitis ranges from 4.9 to 35 per 100,000 population. Acute pancreatitis is the leading cause of hospitalization in the United States among gastrointestinal causes [2]. In a systematic review of studies of acute pancreatitis, overall mortality was 5%, 3%, and 17% in all cases of acute pancreatitis, interstitial, and necrotizing pancreatitis, respectively [3]. Drug-induced pancreatitis is not unknown. We describe one such instance of drug-induced pancreatitis secondary to isotretinoin.

## Case presentation

**Figure 1:**
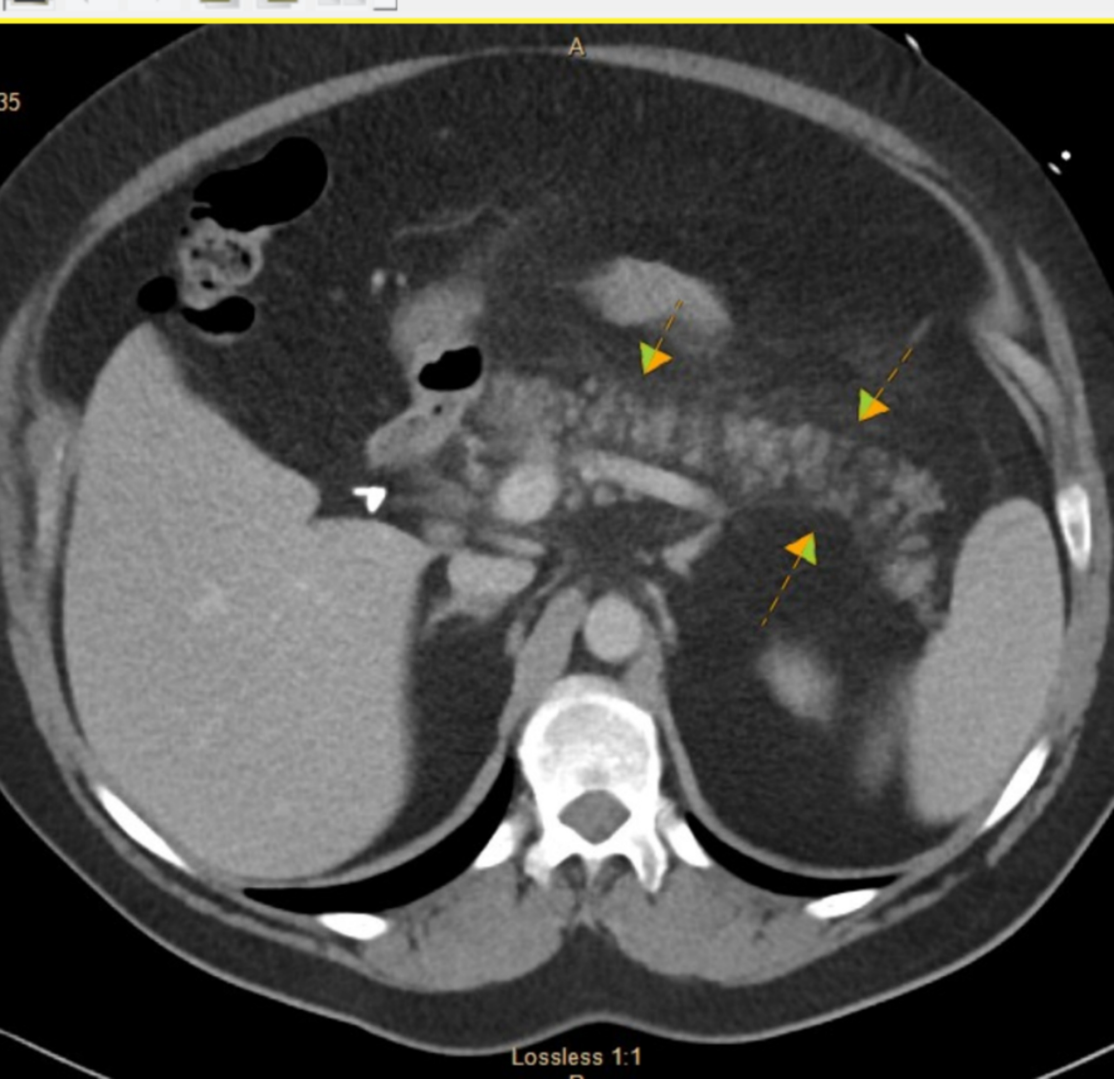
CT scan showing the pancreas appears diffusely edematous and there is some induration of the fat around the pancreas suggestive of mild pancreatitis.

A 55-year-old female with a known past medical history of gastroesophageal reflux disease, hyperlipidemia, acne, depression, and history of cholecystectomy presented to the emergency room with the symptoms of abdominal pain and low-grade fever. The pain was epigastric in location and radiated to her back. The pain was severe in intensity and constant in nature. The patient denied any history of alcoholism, and the serum alcohol level was undetectable. The patient reported no recent history of insect bite. The patient stated that she started taking isotretinoin a month ago, which was prescribed by a dermatologist for severe acne. There were no other new medications or medication changes for the past two years. Vital signs were stable except that the patient had a temperature of 99 degrees Fahrenheit initially, which later went up to 101 degrees Fahrenheit. The patient also had epigastric tenderness on palpation with no peritoneal signs. Workup in the emergency room included CT scan of the abdomen (Figure 1), which showed changes consistent with mild acute pancreatitis. Ultrasound gallbladder showed no gallstones. Laboratory data showed lipase of 1,500 U/l (reference range 73-393 U/l), and serum triglyceride levels of 1,300 mg/dL (reference range 15-149 mg/dL). Complete blood count and comprehensive metabolic panel were unremarkable. The patient had a lipid profile done two years ago, and her triglyceride levels were 160 mg/dL. The patient never had any such episode in the past. The patient was treated with bowel rest, intravenous fluid, and prescribed intravenous narcotic pain medications for pain control. Isotretinoin was discontinued, and the patient was advised not to retake it. The patient improved over the next few days and was discharged home in a stable condition.

## Discussion

Drug-induced pancreatitis is not uncommon. Pancreatitis due to medications is rare (0.3% to 1.4%), although limited data suggest that the incidence may be increasing [4-7]. Several drugs have been associated with drug-induced pancreatitis, and several different mechanisms of drug-induced pancreatitis have been proposed [8,9]. These mechanisms include immunologic reactions (e.g., 6-mercaptopurine, aminosalicylates, sulfonamides), direct toxic effect (e.g., diuretics, sulfonamides), accumulation of a toxic metabolite (valproic acid, didanosine, pentamidine, tetracycline), ischemia (diuretics, azathioprine), intravascular thrombosis (estrogen), and increased viscosity of pancreatic juice (diuretics and steroids).

Isotretinoin has also been seen to cause such episodes in some case reports secondary to hypertriglyceridemia [10-12]. Patients need to be informed of such potential risks associated with this drug at the time of prescription. Fortunately, our patient did not have severe pancreatitis and stopped the medication, and supportive treatment is likely needed in such patients. The prognosis of drug-induced pancreatitis is generally excellent, and mortality is low [13]

## Conclusions

Patients should be informed about the possibility of hypertriglyceridemia-induced pancreatitis while prescribing isotretinoin, and their serum triglycerides should be closely monitored. This case guides clinicians to think outside the box and not fixate and attribute every case of pancreatitis with negative initial workup for common causes to idiopathic. There are many other drugs with such rare side effects, and medication history should always be reviewed in detail.
